# Eutrophication and the dietary promotion of sea turtle tumors

**DOI:** 10.7717/peerj.602

**Published:** 2014-09-30

**Authors:** Kyle S. Van Houtan, Celia M. Smith, Meghan L. Dailer, Migiwa Kawachi

**Affiliations:** 1 NOAA Fisheries, Pacific Islands Fisheries Science Center, Honolulu, HI, USA; 2 Nicholas School of the Environment and Earth Sciences, Duke University, Durham, NC, USA; 3 Department of Botany, University of Hawaiʻi, Honolulu, HI, USA

**Keywords:** Superweeds, Luxury consumption, Invasive species, Wastewater, Coastal ecology, Ecosystem based management, Nutrient pollution, *Chelonia mydas*, Firbropapillomatosis

## Abstract

The tumor-forming disease fibropapillomatosis (FP) has afflicted sea turtle populations for decades with no clear cause. A lineage of *α*-herpesviruses associated with these tumors has existed for millennia, suggesting environmental factors are responsible for its recent epidemiology. In previous work, we described how herpesviruses could cause FP tumors through a metabolic influx of arginine. We demonstrated the disease prevails in chronically eutrophied coastal waters, and that turtles foraging in these sites might consume arginine-enriched macroalgae. Here, we test the idea using High-Performance Liquid Chromatography (HPLC) to describe the amino acid profiles of green turtle (*Chelonia mydas*) tumors and five common forage species of macroalgae from a range of eutrophic states. Tumors were notably elevated in glycine, proline, alanine, arginine, and serine and depleted in lysine when compared to baseline samples. All macroalgae from eutrophic locations had elevated arginine, and all species preferentially stored environmental nitrogen as arginine even at oligotrophic sites. From these results, we estimate adult turtles foraging at eutrophied sites increase their arginine intake 17–26 g daily, up to 14 times the background level. Arginine nitrogen increased with total macroalgae nitrogen and watershed nitrogen, and the invasive rhodophyte *Hypnea musciformis* significantly outperformed all other species in this respect. Our results confirm that eutrophication substantially increases the arginine content of macroalgae, which may metabolically promote latent herpesviruses and cause FP tumors in green turtles.

## Introduction

**Figure 1:**
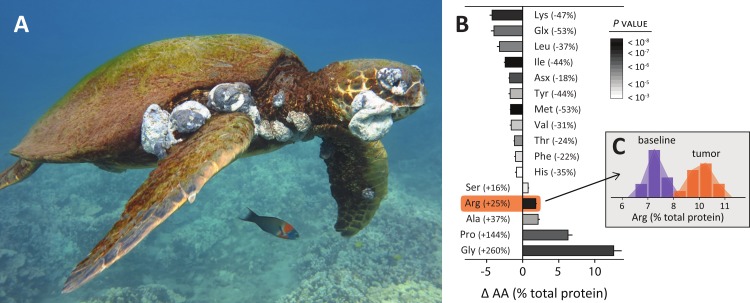
(A) Juvenile green turtle (*Chelonia mydas*) severely afflicted with fibropapillomatosis, a tumor-forming disease associated with *α*-herpesviruses. Photo: August 2012 Makena, Maui (credit: Chris Stankis, Flickr/Bluewavechris). (B) Amino acid profiles from turtle tissues show fibropapilloma tumors are notably enriched in glycine, proline and arginine, and depleted in lysine. Glycine is a known tumor biomarker; proline aids herpesvirus infections; and arginine and lysine promote and inhibit herpesviruses, respectively. Bars represent the average difference between tumor and baseline tissue for 12 individual turtles, percent changes from baseline percent total protein listed in parentheses, error bars are SEM. Bar color indicates *P* values from two-tailed paired *t*-tests. (C) Underlying histograms for arginine content in baseline and tumor tissue samples, bars are raw values, curves are smoothed trend.

Fibropapillomatosis (FP) is a chronic and often lethal tumor-forming disease in sea turtles (Fig. 1A). It became a panzootic in green turtles in the 1980s, prompting concern that it was a serious threat to their global conservation (Chaloupka et al., 2008; Herbst, 1994). Though most green turtle population indices have increased steadily since (Seminoff et al., 2014), the disease remains prevalent and in several locations its incidence is still increasing (Van Houtan, Hargrove & Balazs, 2010). Advances in understanding the cause of FP have recently centered on environmental factors, with diverse lines of evidence from genomics to epidemiology supporting this hypothesis (Aguirre & Lutz, 2004; dos Santos et al., 2010; Ene et al., 2005; Herbst et al., 2004; Van Houtan, Hargrove & Balazs, 2010). The ecological promotion of the disease is made further interesting as FP tumors have a proposed viral origin.

Early studies discovered DNA from *α*-herpesviruses in FP tumors, but found adjacent tissues from diseased turtles, as well as samples from clinically healthy turtles, to be free of herpes DNA (Lackovich et al., 1999; Lu et al., 2000; Quackenbush et al., 1998). Though further progress has been limited by an inability to develop viral cultures, recent work with next generation genomic techniques have made important contributions. These studies (Alfaro-Núñez & Gilbert, 2014; Page-Karjian et al., 2012) found herpesvirus DNA to be rather ubiquitous—occurring in all hard-shelled sea turtles, in all populations tested, and even prevalent in clinically healthy turtles. If *α*-herpesviruses are the origin of FP, this represents a classic herpesvirus scenario where infections are pervasive, but latent or subclinical, in the host population (Stevens & Cook, 1971; Umbach et al., 2008) and revealing of its etymology from the Greek ε*ρπης*, meaning “to creep”. With this in mind, we recently described the epidemiological link between this disease and coastal eutrophication, detailing how green turtles could literally be eating themselves sick (Hall et al., 2007), activating latent herpes infections and promoting tumors by foraging on arginine-enriched macroalgae. A model built on this hypothesis (Van Houtan, Hargrove & Balazs, 2010) explained 72% of the spatial variability of the disease across the Hawaiian Islands while offering a detailed explanation of the disease that connects turtle ecology, plant physiology (e.g., Raven & Taylor, 2003), and herpes biology to known management problems of nutrient pollution and invasive species.

At the forefront, this proposed pathway focuses on the role arginine might have in promoting FP tumors. A significant body of evidence supports this. In many chronic diseases, arginine is implicated in cell inflammation and immune dysfunction (Peranzoni et al., 2008) and in promoting viral tumors (Mannick et al., 1994). But arginine is specifically important for herpesviruses. Laboratory studies demonstrate that herpes infections require arginine, being stunted in its absence (Inglis, 1968; Mikami, Onuma & Hayashi, 1974; Olshevsky & Becker, 1970) and diminished when it is deprived (Mistry et al., 2001). Subsequent research revealed that arginine is a principal component of glycoproteins in the outer viral envelope of herpesviruses. These glycoproteins are conserved across a wide variety of herpesviruses (Alfaro-Núñez, 2014) and are critical to the herpes life cycle as they facilitate localization, fusion, and entrance to host cell nuclei (Hibbard & Sandri-Goldin, 1995; Klyachkin & Geraghty, 2008). Beyond its significance for herpesviruses, arginine is an emerging focus of human cancer treatments as well. Cancer tumors lacking enzymes that synthesize arginine must obtain arginine metabolically, and can therefore be regulated by arginine deprivation (Bowles et al., 2008; Feun et al., 2008; Kim et al., 2009).

Perhaps coincidentally, arginine also plays an important role in how plants sequester environmental nitrogen. Nitrogen is a limiting factor for both plant and macroalgal growth (Raven & Taylor, 2003). As a result, in times of environmental availability plants acquire excess nitrogen through what is known as luxury consumption (Chapin III, 1980). Terrestrial plants, however, do not rely on a host of amino acids for luxury consumption; they preferentially store ambient nitrogen in arginine (Chapin III, 1980; Chapin III, Schulze & Mooney, 1990; Llàcer, Fita & Rubio, 2008). Little is known about how this functions in macroalgae, however. Previous studies in Hawaii suggest it might be relevant. Macroalgae from these limited surveys demonstrated that amino acids and stable isotope values for *δ*^15^N varied by species and by location (Dailer et al., 2010; McDermid, Stuercke & Balazs, 2007). Though the data were limited, arginine was specifically elevated at eutrophic sites for two invasive species of *Ulva* and *Hypnea* (McDermid, Stuercke & Balazs, 2007), prompting more systematic study.

Eutrophication of coastal waters in Hawaii has spurred chronic nuisance algal blooms and dramatically altered the composition of reef ecosystems (Cox et al., 2013; Dailer et al., 2010; Lapointe & Bedford, 2011; Smith, Hunter & Smith, 2010). Non-native macroalgae introduced across the Main Hawaiian Islands after 1950 have been particularly influential (Abbot, 1999; Smith, Hunter & Smith, 2002), having displaced native algae and become the dominant forage for Hawaiian green turtles (Russell & Balazs, 2009). Despite the emergence of FP, and historical overharvesting (Kittinger et al., 2013; Van Houtan & Kittinger, 2014), numbers of nesting green turtles have grown steadily in Hawaii since their protection under state and federal regulations in the 1970s (Seminoff et al., 2014). Nonetheless, FP remains the greatest known mortality to Hawaiian green turtles (Chaloupka et al., 2008) and in some regions the incidence of FP tumors is still on the rise (Van Houtan, Hargrove & Balazs, 2010). Beyond its influence on green turtle populations, eutrophication is also associated with coral reef declines (Vega Thurber et al., 2014). Growth anomalies in *Porites* corals, for example, occur in the same eutrophied Hawaii reefs as diseased green turtles (Friedlander et al., 2008), and these coral tumors have *Herpesviridae* gene signatures (Vega Thurber et al., 2008). Understanding the promotion of FP tumors may therefore be broadly relevant for the conservation of coral reef ecosystems.

Here we analyze tissues from tumored green turtles and dominant forage species of macroalgae from across Hawaii. We determine amino acid content using High-Performance Liquid Chromatography (HPLC) to establish tumor biomarkers (Jain et al., 2012) and examine nutrient changes in macroalgae and luxury consumption. We run a series of Generalized Linear Models (GLMs) to test for arginine levels, arginine storage, and to examine the role of eutrophication. Collectively, these analyses are an interdisciplinary test of a hypothesis we posed earlier (Van Houtan, Hargrove & Balazs, 2010; Van Houtan & Schwaab, 2013) that arginine would be elevated in invasive algae in eutrophic locations and this would in turn promote FP tumors in green turtles.

## Materials and Methods

### Tissue collection and preparation

We sampled turtle tissues during necropsies of stranded green turtles at the NOAA Pacific Islands Fisheries Science Center in Honolulu (US FWS permit # TE-72088A-0). For turtles with heavy tumor burdens, we collected both tumor and baseline tissue. Using a scalpel to make radial cross-sections, we obtained at minimum 0.5 cm^3^ for each tissue type, selecting subsurface material to avoid contamination. Tumors were sampled from the flipper or eye, and baseline tissue was subcutaneous muscle from the flipper or pectoral that appeared grossly subclinical. We collected samples from 12 turtles, representing males and females from a variety of life stages (Table S1 provides full details). At collection, we rinsed samples in water and stored in 90% alcohol in 1.5 mL cryovials (Thermo Scientific™ Nalgene™). After 24–48 h, we pressed the samples dry with forceps, and transferred to clean cryovials packed with SiO_2_ indicating gel desiccant (Fisher™, grade 48, 4–10 mesh). We replaced the spent silica beads every 24 h, repeating the process as needed until the samples were completely dried. We homogenized the resulting tissues with a porcelain mortar and pestle or by shaving samples with a #22 scalpel blade.

We collected five species of macroalgae from coastal watersheds spanning a range of nutrient profiles (Van Houtan, Hargrove & Balazs, 2010) on Oahu, Maui, and Hawaii island. We focused on three invasive species—*Hypnea musciformis* (Wulfen) JV Lamouroux, *Acanthophora spicifera*(M Vahl) Borgesen, and *Ulva lactuca* Linnaeus—and two non-invasive native species—*Pterocladiella capillacea* (SG Gmelin) Santelices & Hommersand and *Amansia glomerata* C Agardh—that are representative turtle forage items (Russell & Balazs, 2009) and reasonably widespread. *P. capillacea* and *A. glomerata* were inconsistently found and combined into a single category. *U. lactuca*, the only chlorophyte, was recently reclassified (O’Kelly et al., 2010) from *U. fasciata*. For each species and location we collected three replicates at 0–5 m depth, where nearshore green turtles commonly forage (Van Houtan & Kittinger, 2014). We rinsed samples with deionized water and later dried in a 60 °C oven (Dailer et al., 2010). We homogenized dried samples with a mortar and pestle and stored in 5 mL cryovials (Thermo Scientific™ Nalgene™). When samples were difficult to obtain, we supplemented our samples with results from a published study in Hawaii (McDermid, Stuercke & Balazs, 2007). Online Supplemental Information provides full sample metadata.

### Amino acid and statistical analysis

We sent prepared samples to the Protein Chemistry Laboratory at Texas A&M University for amino acid determination. Samples were separated into three aliquots, weighed, and then placed in 200 µL of 6N HCl along with the Internal Standard and hydrolyzed at 110 °C for 22 h. The resulting amino acids were separated and quantified using an HPLC (Agilent™ 1260) with pre-column derivitization (Blankenship et al., 1989) by ortho-phthalaldehyde (OPA) and fluorenylmethyl chloroformate (FMOC). As both tryptophan and cysteine are destroyed during hydrolysis, the total protein measured is slightly underreported. As a result, this analysis reports the dry mass for 16 amino acids (Fig. 1B), which we calculate as the mass divided by the total sample mass, averaged between sample aliquot replicates.

For turtle tissues, we calculate the change of amino acids from the baseline to tumor samples, expressed as the difference of the average percent total protein for each tissue type. For macroalgae samples, we first calculate the change in arginine levels in samples from eutrophic and oligotrophic sites. Collection sites were considered eutrophic if they had a nitrogen footprint statistic (Van Houtan, Hargrove & Balazs, 2010) above 0.50 and oligotrophic if not. (There was a clear separation here as no sites fell between 0.37 and 0.57.) Sites straddling two watersheds were also located on remote geographic peninsulas with little human impacts (Van Houtan, Hargrove & Balazs, 2010) and therefore given the lower of the two watershed footprint statistics. To describe how macroalgae store environmental nitrogen, we multiplied the amino acid dry mass (percent of total) for each sample by the proportional molecular weight of nitrogen.

We formally test for statistical sample differences through a variety of generalized linear models (GLM). To assess amino acid changes between tumors and turtle baseline tissue, we run a paired *t*-test for sample means and plot the results to identify potential biomarkers (Jain et al., 2012). To examine arginine variability of individual algae across site types we use a one-tailed *t*-test. We follow this with a GLM that has site treatment and species as factors to predict arginine content. As a frame of reference, we combine these results with known energetic requirements of green turtles (Jones et al., 2005) and published energy content of alga in our study (McDermid, Stuercke & Balazs, 2007) to estimate the daily arginine intake. We calculate this for subadult turtles, the highest-diseased demographic in Hawaii (Van Houtan, Hargrove & Balazs, 2010), as well as for large adults, for different site-species comparisons. To test for luxury consumption, we fit normal distributions to the observed nitrogen amino acid dry mass values for each sample, and determine if arginine falls outside the distribution’s expected 95% interval. We then examine the cause of arginine nitrogen variability. We first build a GLM with total plant nitrogen and species as factors, and then a second with nitrogen footprint and species as factors.

## Results

Figure 1B plots the amino acid profiles of tumor-baseline tissue sets for 12 green turtles. We observed significant differences in all 16 amino acids tested, highlighting the divergent metabolism of tumors. Methionine (tumor depleted) and arginine (tumor enriched) had the most statistically significant changes. Glycine, however, had the most dramatic shift. Tumor glycine increased on average 260% (range 93–382%, *t* = 11.4, *P* < 0.0001), meaning tumors had 2–5 times more glycine than baseline tissues. This is perhaps unsurprising as glycine is a building block for nucleic acids and is required in large amounts by rapidly proliferating cancer cells (Jain et al., 2012; Tomita & Kami, 2012). Proline also increased markedly in tumors (average 144%, range 40–269%, *t* = 10.1, *P* < 0.0001), the second largest change we observed. This may reflect the importance of proline for herpesviruses in counteracting host cell defenses. The herpesvirus protein *Us11* has an arginine- and proline-rich binding domain that specifically inhibits PKR (protein kinase R), critical for cellular viral defense (Khoo, Perez & Mohr, 2002; Poppers et al., 2000). Proline synthesis was also important in recent analyses of cancer tumors (De Ingeniis et al., 2012; Nilsson et al., 2014).

Arginine increased (average 25%, range 9–38%, *t* = 12.9, *P* < 0.0001) and lysine decreased (range: average 47%, range 23–67%, *t* = −12.1, *P* < 0.0001) in tumors, consistent with their respective demonstrated roles in herpes infections (Fatahzadeh & Schwartz, 2007; Griffith et al., 1987; Hibbard & Sandri-Goldin, 1995; Inglis, 1968; Mikami, Onuma & Hayashi, 1974; Olshevsky & Becker, 1970). Figure 1C plots the underlying raw histograms for arginine percent total protein in baseline samples and tumors, with smoothed distributions in the background. Aside from the above results, tumors were depleted in glutamine (53%), leucine (37%), isoleucine (44%), asparagine (18%), tyrosine (44%), valine (31%), threonine (24%), phenylalanine (22%), and histidine (35%)—listed in order of percent total protein change. Tumors were also enriched in alanine (37%) and serine (16%), the latter being essential in breast cancers (De Ingeniis et al., 2012; Possemato et al., 2011). These amino acid profiles serve as a first template for establishing FP biomarkers that may aid understanding this disease, and for herpesviruses and tumor formation more generally.

**Figure 2:**
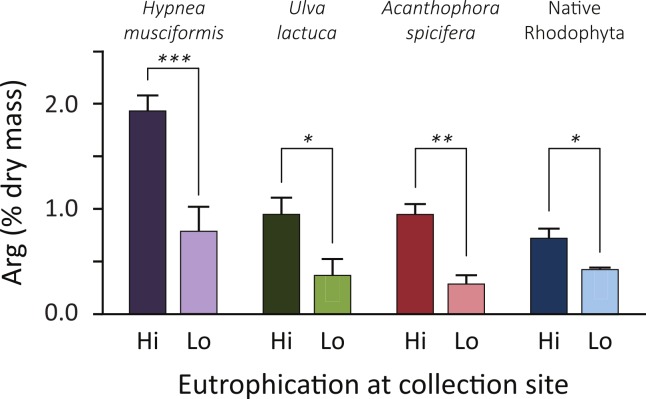
Common forage species for green turtles are arginine enriched at eutrophic coastal areas. Arginine content is 2–3 times higher at eutrophic sites, compared to the same alga sampled at oligotrophic sites. Increases are more pronounced, and arginine levels are higher, in the three nonnative invasive macroalgae species than for the two native Rhodophyta, *Amansia glomerata* and *Pterocladiella capillacea*. Error bars indicate SEM. Asterisks are one-tailed *t*-test results: ^∗^*P* < 0.05; ^∗∗^*P* = 0.01; ^∗∗∗^*P* < 0.01. A GLM with site treatment and species as factors to predict arg content is statistically significant (*F*_(7,24)_ = 11.8, *P* < 0.0001, *R* = 0.91). Given energetic estimates, green turtles foraging on non-native algae at eutrophied sites can increase their daily arginine intake by 17–26 g.

Analyzing forage, Fig. 2 plots the arginine enrichment in common forage species of wild macroalgae between locations of low and high nutrient inputs. Arginine levels increased at eutrophic sites in all species (average 160%, range: 70–230%). Though *A. spicifera* had the highest increase (230%, *t* = 3.5, *P* = 0.01), *H. musciformis* had the highest arginine content at eutrophic (1.94% dry mass) and oligotrophic (0.79% dry mass) sites and the most statistically significant change (*t* = 4.1, *P* = 0.007). Of note, the *H. musciformis* arginine content at oligotrophic sites was higher than that of native rhodophytes sampled at eutrophic sites (0.73% dry mass). This may highlight the role of aggressively invasive macroalgae (Smith, Hunter & Smith, 2002) such as *H. musciformis* in this disease. A more complete GLM with site treatment and species as factors predicts arginine content (*F*_(7,24)_ = 11.8, *P* < 0.0001, *R* = 0.91).

But this does not quite capture the nutrient intake of turtles foraging at different site treatments. From energetics we know a 45 kg subadult green turtle requires 2,435 kJ day^-1^ of dietary energy (Jones et al., 2005). If this turtle only foraged on *H. musciformis*—with an energy content of 4.3 kJ g^-1^ total dry mass (McDermid, Stuercke & Balazs, 2007)—it would require 567 g dry mass of *H. musciformis* daily to meet energetic demands. Based on our amino acid analysis (Fig. 2) this turtle would consume 11.1 g of arginine daily at eutrophic sites. If this same turtle only consumed the native species we tested (*P. capillacea* and *A. glomerata*, average energy 8.9 kJ g^-1^ total dry mass (McDermid, Stuercke & Balazs, 2007)) it would require 274 g dry mass of daily forage. At oligotrophic sites this turtle would consume 1.2 g arginine per day. In other words, foraging on invasive alga, *H. musciformis*, at eutrophic sites increases the average arginine intake by 9.9 g day^-1^ (range 7.8–11.8 g) by comparison to consuming native species at oligotrophic sites, which is 5–14 times the baseline arginine consumption. If we consider this for a 100 kg adult turtle requiring 5,364 kJ daily (Jones et al., 2005) the arginine boost is 21.8 g day^-1^ (range 17.2–26.0 g). Clearly, there is a substantial dietary influx of arginine for green turtles foraging in eutrophied watersheds.

**Figure 3:**
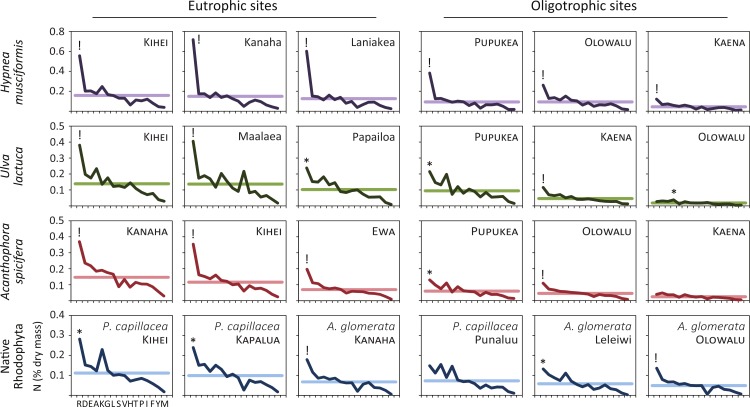
Like terrestrial plants, macroalgae preferentially sequester available environmental nitrogen in arginine. We quantify this luxury consumption by calculating the nitrogen dry mass in each amino acid across species and ecosystem treatments. Horizontal lines are the mean nitrogen dry mass for each sample, **!** indicates value lies outside the expected 99% interval, and ∗ outside the expected 95% interval. All (13/13) of the eutrophic and 75% (9/12) of the oligotrophic site samples show preferential sequestration of nitrogen in arginine. Amino acids are arranged from left to right in average descending order of prevalence: arginine, R; aspartic acid, D; glutamic acid, E; alanine, A; lysine, K; glycine, G; leucine, L; serine, S; valine, V; histidine, H; threonine, T; proline, P; isoleucine, I; phenylalanine, F; tyrosine, Y; methionine, M.

Figure 3 plots the nitrogen dry mass for each amino acid to examine how macroalgae sequester environmental nitrogen. In 13/13 samples from eutrophic sites and 9/12 (75%) samples from oligotrophic sites, nitrogen levels were anomalously high for arginine. That is, nitrogen dry mass was outside the expected 95% interval set by the fitted normal distribution parameters for that sample. Two samples from oligotrophic sites (Punaluu *P. capillacea* and Kaena *A. spicifera*) demonstrated no preferential nitrogen storage. One sample (Olowalu *U. lactuca*) had a positive anomaly for alanine, but its total nitrogen levels were the lowest measured, minimizing its significance. We generated Fig. 3 before receiving the results for a seventh *Ulva* sample (eutrophic Kanaha). This panel appears in the online supplement.

Though arginine was the clear preference for nitrogen storage (22/25 total samples, 88%), this was often extreme. In 15 samples (denoted by “!” in Fig. 3) arginine nitrogen storage is outside the 99% interval (above the expected 99.5% cumulative probability distribution) for that sample. Such extreme arginine preference occurred in all *H. musciformis* samples, followed by *A. spicifera* (4/6 samples, 67%), *U. lactuca* (3/7 samples, 43%), and the native rhodophytes (2/6 samples, 33%)—and was observed in 9/13 (69%) samples from eutrophic sites considering all species. Thus, the marine macroalgae we sampled demonstrated a clear tendency to sequester environmental nitrogen as arginine, which is an interesting convergence with terrestrial plants (Chapin III, 1980; Chapin III, Schulze & Mooney, 1990).

**Figure 4:**
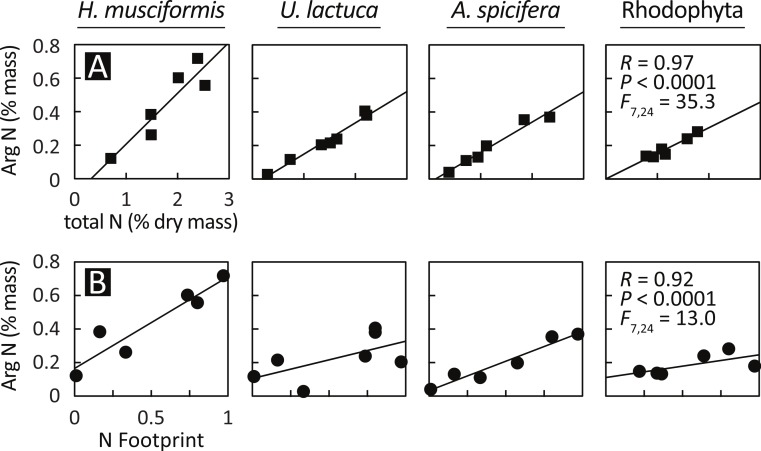
Arginine sequestration of environmental nitrogen increases with (A) total plant N and (B) in proportion with watershed eutrophication. A GLM with total plant nitrogen and species as factors is statistically significant (*F*_(7,24)_ = 35.3, *P* < 0.0001, *R* = 0.97), as is a model with nitrogen footprint (Van Houtan, Hargrove & Balazs, 2010) and species as factors (*F*_(7,24)_ = 13.0, *P* < 0.0001, *R* = 0.92). The relationships are similar between species, except for *H. musciformis*, which has a steeper slope in both panels, indicating *H. musciformis* is more efficient at sequestering environmental nitrogen and in storing it in arginine.

Having documented elevated arginine at eutrophic sites and arginine luxury consumption, Fig. 4 assesses the relationship between arginine nitrogen storage, total tissue nitrogen, and watershed-level eutrophication metrics. Arginine nitrogen increased with total tissue nitrogen for all species (Fig. 4A), and a GLM with total tissue nitrogen and species as factors is highly significant (*F*_(7,24)_ = 35.3, *P* < 0.0001, *R* = 0.97). Figure 4B shows that arginine nitrogen also increased with each site’s nitrogen footprint (Van Houtan, Hargrove & Balazs, 2010); an index of natural and anthropogenic factors that generate, deliver, and retain nitrogen in coastal watersheds, and a proxy for local nitrogen loading. Similar to the previous model, a GLM with nitrogen footprint and species as factors is significant (*F*_(7,24)_ = 13.0, *P* < 0.0001, *R* = 0.92).

The relationships for these models are similar between species, except for *H. musciformis*, which has a steeper slope in both panels. In Fig. 4A, this indicates that given environmental levels, *H. musciformis* allocates proportionally more nitrogen to arginine than other amino acids, for the alga tested. Figure 4B suggests *H. musciformis* is more proficient at sequestering environmental nitrogen in arginine than the other species. Similar to Fig. 2, this perhaps underscores the importance of *H. musciformis* in promoting FP tumors. Though the arginine levels (Fig. 2) and the arginine nitrogen storage (Fig. 3) are not as high in the native macroalgae as for *U. lactuca* and *A. spicifera*, the statistical relationships between arginine nitrogen, tissue nitrogen, and ecosystem nitrogen are similar (Fig. 4).

## Discussion

In this study we demonstrated how eutrophication increases the arginine in invasive marine macroalgae; that this significantly boosts the arginine intake by foraging green turtles, and that arginine is elevated in tumors from diseased green turtles. Based on energetic needs, we calculated adult turtles foraging in eutrophic habitats on invasive algae might boost their arginine intake 5–14 times, consuming a total of 22–28 g of arginine daily. We provide a first baseline set of amino acid biomarkers for FP tumors, and we documented arginine luxury consumption across a variety of macroalgae. We discuss the results and their implications for understanding the disease and its environmental promotion below.

Though FP tumors were elevated in several amino acids, dietary shifts in arginine may be significant. Glycine, proline, alanine, arginine and serine all were elevated in tumors (Figs. 1B–1C). Of these amino acids, glycine (Jain et al., 2012), proline (De Ingeniis et al., 2012; Nilsson et al., 2014), and serine (Possemato et al., 2011) are known tumor biomarkers; where arginine and proline have added significance for herpesviruses. Of the elevated amino acids, however, only arginine increased in macroalgae at eutrophied sites (where disease rates are elevated) and has a functional role in nitrogen luxury consumption (Fig. 3). Arginine nitrogen content, for example, was above the 95% expected interval in 88% of our macroalgae samples, and was extreme (above the 99% interval) in 60% of our samples (Fig. 3).

This suggests arginine may be the critical ingredient linking nearshore eutrophication, luxury consumption, turtle diet, and FP tumors. The metabolic pathways here are uncertain, however. Metabolic reprogramming is a hallmark of rapidly proliferating cancer cells, and a growing body of literature has focused on carbon metabolism in tumors (De Ingeniis et al., 2012; Jain et al., 2012; Nilsson et al., 2014). Perhaps it is unsurprising that we detected significant tumor-baseline differences for all 16 amino acids. However, instead of focusing on carbon metabolism common to cancer studies, we profiled nitrogen due to its role in limiting macroalgae growth. Future progress in understanding FP may therefore come from a systematic characterization of the metabolic pathways in FP tumors, and in particular the recycling, salvaging, and biosynthesis of arginine. A dietary role for tumor promotion in human cancers may also benefit from a more comprehensive understanding of nitrogen metabolism.

Our results help explain the epidemiology of this disease, and highlight the role of environmental factors in Hawaii and perhaps beyond. Though DNA from herpesviruses linked to FP tumors is found in all sea turtle species, this disease has only been widespread and a conservation concern for green turtles (Alfaro-Núñez, 2014). This is consistent with our proposed mechanism involving eutrophication and arginine intake. Green turtles are the only strictly herbivorous sea turtle and therefore would consume the most arginine-enriched algae in nearshore habitats (other omnivorous sea turtle species consume algae, though at lower rates). The spatial and demographic structure of green turtles may also be relevant. Juvenile green turtles have a pelagic phase until they recruit to nearshore habitats as young juveniles (Seminoff et al., 2014). Far away from human population centers, green turtles are disease-free during this pelagic phase (Ene et al., 2005; Van Houtan, Hargrove & Balazs, 2010). In the Main Hawaiian Islands (MHI), the incidence of FP tumors increases steadily as turtles mature, and then decreases when they begin migrating to the relatively pristine Northwestern Hawaiian Islands to breed. In other words, disease rates increase directly in proportion to their residency time in the Main Hawaiian Islands (Van Houtan, Hargrove & Balazs, 2010). In addition to this chronic exposure to eutrophic habitats, older turtles have greater energetic demands and therefore may additionally have higher disease rates due to increases in their consumption of MHI macroalgae and subsequent arginine intake. Though we are investigating environmental influences, it is possible that immunocompetence could factor in these patterns, but its influence is unknown.

Aside from demographic patterns in turtles, invasive species of macroalgae also seem to be influential. Certain regions—such as the Kona coast of Hawaii island—have curiously low disease rates. While we previously demonstrated that this region has few nutrient inputs and invasive algae are uncommon (Van Houtan, Hargrove & Balazs, 2010), our results here help explain this pattern. In this study we showed foraging green turtles could be more easily satiated by native macroalgae, as they can have relatively higher energy contents. Combined with our amino acid results, the energy and arginine content of macroalgae may therefore act as a sort of one-two punch for promoting this disease. Native macroalgae have a fraction of the arginine content of invasive species (Fig. 2), but offer more calories per unit mass (see online Supplemental Information). Turtles foraging on invasive macroalgae in eutrophic areas would need twice the amount they would require of native algae, therefore multiplying the arginine enrichment effect. For so-called superweeds like *H. musciformis*, this low energy–high arginine combination is the most extreme we observed. Considering that *H. musciformis* energy content can vary inversely with growth rate (Guist Jr, Dawes & Castle, 1982), this may be a general result, and a topic for future research.

Our estimates for turtle arginine consumption were often substantial, but the numbers could be even higher. For subadults we documented an average increase of 9.9 g arginine day^-1^ when shifting from native forage at oligotrophic sites to invasive forage at eutrophic sites. This number jumped to an average 21.8 g day^-1^ for adults. These numbers are based on our observed amino acid values and energetic demands. The metabolic rates we reference are baseline averages (Jones et al., 2005), which would underestimate the dietary needs of rapidly growing turtles, migrating animals, or adult females amassing resources for vitellogenesis. Our calculated dietary intake of arginine could therefore increase, making the already significant increase even more so. There are no daily nutritional guides for wild green turtles (Bjorndal, 1997). However, to put these numbers in context, they are well above the recommended dietary allowance for humans. Human adults (19–50 years) should consume 4.7 g of arginine and 51 g of total protein daily (Institute of Medicine, 2005). Our estimated arginine intake for adult turtles could reach 28 g day^-1^ (online Supplemental Information), which is half the recommended total daily protein for humans and 5 times the suggested arginine intake. Future studies can use our arginine intake estimates to guide treatments of turtles with FP tumors.

Across green turtle populations, it is widely observed that FP occurs most frequently in eutrophied and otherwise impaired waterways (Herbst, 1994; Van Houtan, Hargrove & Balazs, 2010). Our efforts here have largely been to demonstrate why this might occur, and to detail the ecological mechanisms. A logical next step is to repeat this study comparing tumors and forage items for other green turtle populations and other species. Additional next steps could be developing a monitoring plan to assess ecosystem risk for the disease in Hawaii and other ecological regions. Stable isotope analyses of tissues from *U. lactuca* or *H. musciformis* are an effective method for monitoring water quality in an integrative manner. For example, for macroalgae that uptake nitrogen from the water column, *δ*^15^N values above 6.0 point to a significant wastewater presence (Dailer et al., 2010; Dailer, Smith & Smith, 2012). While these tests can reveal plant nitrogen sources, they do not comment on ecosystem nitrogen flux. Our test for nitrogen arginine sequestration (Fig. 3) may infer on nitrogen flux at a more informative level than total tissue nitrogen, however, more research here is necessary. Combined stable isotope and amino acid analysis of macroalgae, therefore could be a powerful and reasonably inexpensive tool ($120 for both tests) to monitor and understand eutrophication in coastal ecosystems. The relevance of this tool extends beyond turtle diseases, but to ecosystem based management of coral reefs, estuaries, and seagrass systems.

## Supplemental Information

10.7717/peerj.602/supp-1 Table S1 Online supplemental material This table provides the full metadata and amino acid results for the algae samples considered in this study. Table 2: This table provides full sample metadata for the 12 turtles from which 24 tissue samples were taken.
