# Supplementary material for: Eutrophication and the dietary promotion of sea turtle tumors
Source: PeerJ. 2014 Sep 30;2:e602. doi: 10.7717/peerj.602 (PMC4184234; doi:10.7717/peerj.602)
Supplement: Table S1 — This table provides the full metadata and amino acid results for the algae samples considered in this study. Table 2: This table provides full sample metadata for the 12 turtles from which 24 tissue samples were taken. [file peerj-02-602-s001.docx]

SUPPLEMENTAL INFORMATION FOR VAN HOUTAN *ET AL “*Eutrophication and the dietary promotion of sea turtle tumors*”*


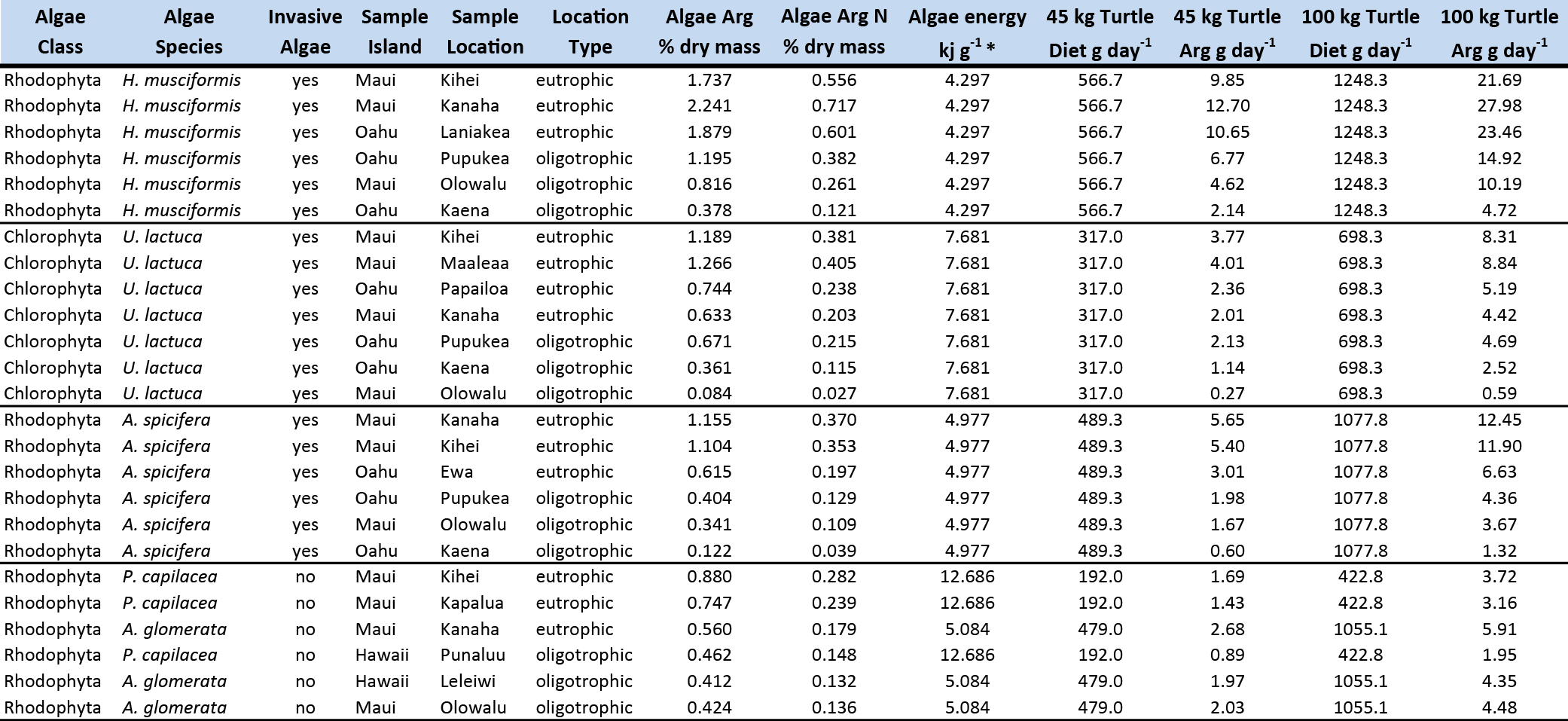


*Supplemental Table 1.*

This table provides the full metadata and amino acid results for the algae samples considered in this study. Samples are organized by class and species, providing biogeographic information, as well as sample location and site treatment. We also list the calculations for energetic requirements and the resulting anticipated arginine intakes discussed in the manuscript text. Asterisk in the column heading indicates values calculated from McDermid et al. 2007. Please see the Methods section for more details.

*Supplemental Table 2*

This table provides full sample metadata for the 12 turtles from which 24 tissue samples were taken. All turtles are green turtles (*Chelonia mydas*) from Oahu, Maui, and Hawaii islands. Turtles come from all demographic groups, as evidenced in the SCL (straight carapace length) sizes listed. Tumor score ranges from 0 (no tumors) to 3 (heavily burdened) as per Chaloupka et al. 2008.
